# Eculizumab for the Treatment of Severe Antibody-Mediated Rejection: A Case Report and Review of the Literature

**DOI:** 10.1155/2016/9874261

**Published:** 2016-07-10

**Authors:** Duy Tran, Anne Boucher, Suzon Collette, Alexis Payette, Virginie Royal, Lynne Senécal

**Affiliations:** ^1^Department of Nephrology, Maisonneuve-Rosemont Hospital, Montreal, QC, Canada H1T 2M4; ^2^Department of Pathology, Maisonneuve-Rosemont Hospital, Montreal, QC, Canada H1T 2M4

## Abstract

In renal transplantation, treatment options for antibody-mediated rejection are limited. Here, we report a case of severe AMR treated with eculizumab. A 50-year-old woman known for end stage kidney disease secondary to IgA nephropathy received a kidney transplant from a 50-year-old deceased donor. At 5 months after transplantation, she presented with acute graft dysfunction and biopsy showed a severe antibody-mediated rejection associated with thrombotic microangiopathy. Despite an aggressive conventional immunosuppressive regimen, signs of rejection persisted and the patient was treated with 3 doses of eculizumab. Following the therapy, markers of TMA improved and graft function stabilized. However, ongoing signs of rejection remained in the repeated biopsy. In kidney transplantation, eculizumab is an expensive treatment and its role in the treatment of antibody-mediated rejection remains to be determined.

## 1. Background

The management of antibody-mediated rejection (AMR) represents a challenge in kidney transplantation, as the treatment options are limited and sometimes ineffective [1]. Here, we report a case of severe AMR treated with eculizumab.

## 2. Case

A 50-year-old Asian woman known for end stage kidney disease secondary to IgA nephropathy received a kidney transplant from a 50-year-old deceased donor. The pretransplant crossmatch (CDC) was negative despite a PRA value of 25% and a complete HLA mismatch (in B and DR). The cold ischemia time was 7 hours and the patient was induced with basiliximab and methylprednisolone. Subsequent maintenance immunosuppression consisted of tacrolimus, mycophenolate, and prednisone.

After transplant, the recipient had delayed graft function requiring three dialysis sessions. Ultimately, the clinical course was favorable and she was discharged with a good renal function (creatinine value of 128 *μ*mol/L (1.48 mg/dL)).

One week after discharge, the patient was readmitted for graft dysfunction with a rise in creatinine to 194 *μ*mol/L. An urgent biopsy showed moderate glomerulitis and the presence of inflammatory cells in peritubular capillaries, but C4d was negative. As C4d-negative antibody-mediated rejection (AMR) was not a recognized entity in 2013; she was only treated with intravenous pulses of methylprednisolone and the creatinine stabilized at 160 *μ*mol/L. A few weeks later, an analysis revealed the presence of a donor specific antibody (DSA) (DQA0302) with MFI of 1800. This DSA was present before the transplant (MFI 1600) but we were not aware of its presence at the time of transplant. The DSA titers decreased mildly after initial treatment (MFI 900).

**Figure 1:**
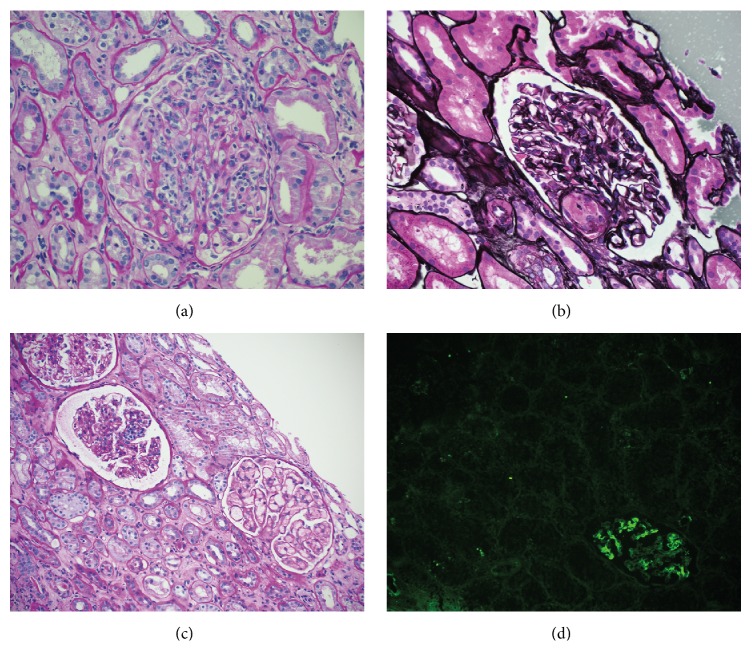
Kidney biopsy findings: (a) transplant glomerulitis with infiltrating mononuclear inflammatory cells within the capillary loops, second biopsy (PAS, magnification ×200); (b) glomerulus with a thrombus involving the vascular pole, second biopsy (Jones, magnification ×200); (c) glomeruli showing persistent transplant glomerulitis and thrombotic microangiopathy, fourth biopsy (PAS, magnification ×100); (d) immunofluorescence microscopy showing C4d mesangial deposition without peritubular capillary staining (magnification ×100).

At 3 months after transplantation, despite a CMV infection treated with ganciclovir, renal function remained stable. However, after 5 months, the recipient presented again with severe acute graft dysfunction (creatinine up to 400 *μ*mol/L) but, this time, there were signs of thrombotic microangiopathy (TMA): thrombocytopenia, decreased haptoglobin, increased LDH, decreased fibrinogen, and hemolytic anemia. A second biopsy was performed and it showed an acute active type 2 AMR with lesions of TMA, peritubular capillaritis, and glomerulitis (negative Cd4) associated with a grade IA cellular rejection (Figure 1). Immunohistochemistry for CMV was negative. She was then treated aggressively with a combination of pulse steroid therapy (3 doses), thymoglobulin (2 doses), plasmapheresis (3 exchanges), intravenous immune globulin (4 doses), and rituximab (4 doses).

**Figure 2:**
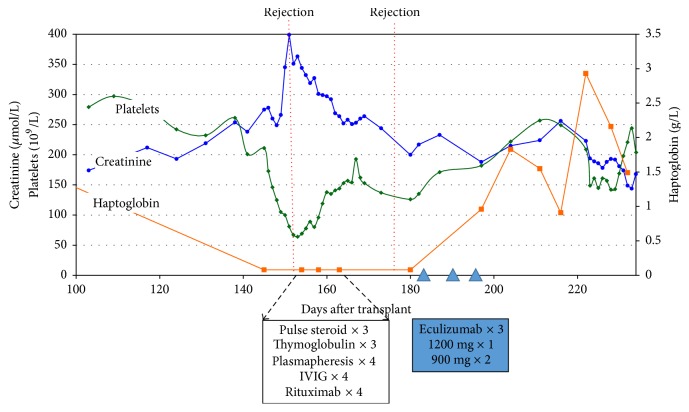
Graft function and microangiopathy markers.

Two weeks after the intensive treatment, despite a stabilization of renal function (creatinine of 200 *μ*mol/L), signs of AMR (TMA) still persisted in a repeat biopsy (3rd biopsy) and DSA remained elevated (MFI 1300). We decided to treat the recipient with 3 doses of eculizumab (1200 mg, 900 mg, and 900 mg) (Figure 1). Following the therapy, blood markers of TMA significantly improved (Figure 2). However, because of the heavy immunosuppressive state, the patient suffered from multiple infections: CMV reactivation, pulmonary aspergillosis, and* Clostridium difficile* colitis. DSA titers were not repeated after eculizumab treatment.

The last graft biopsy (8 months after transplant) showed ongoing AMR and progression of chronic lesions (moderate tubular atrophy and interstitial fibrosis) (Figure 1). However, transplant glomerulopathy and peritubular capillary basement membrane multilayering were not seen in the electron microscopy (EM). One year after transplant, renal function declined progressively (creatinine up to 250 *μ*mol/L) and haptoglobin gradually decreased, but platelets were normal and there was only mild anemia. All infectious complications resolved.

## 3. Discussion

In AMR, the complement system is activated and constitutes one of the main mechanisms responsible for endothelial damage. Eculizumab is a monoclonal antibody that specifically binds to factor C5 and inhibits the terminal pathway of the complement cascade, preventing endothelial injury [2]. Clinically, it is mainly used for the treatment of paroxysmal nocturnal hemoglobinuria and atypical hemolytic uremic syndrome. In renal transplantation, there are few data on the use of eculizumab for the treatment of AMR.

In our case, the diagnosis of AMR was not done initially despite the presence of glomerulitis and peritubular capillaritis because C4d-negative AMR was not a recognized entity before 2014. Therefore, the patient was not aggressively treated after the first biopsy. Once the diagnosis of AMR was made, she had CMV infection that limited the immunosuppressive treatment. However, she probably could have benefited from a more aggressive and longer conventional treatment. By the time eculizumab was introduced, it was probably too late as AMR had become extremely severe. Furthermore, because eculizumab is an expensive medication and not officially approved for AMR treatment, the patient only received 3 doses. Despite improved TMA blood parameters and transient stabilization of renal function following the use of eculizumab, signs of severe AMR persisted in the biopsies. The overall treatment failed to decrease the titer of DSA and because of the heavy immunosuppressive burden she developed several potentially fatal infections.

Stegall et al. used eculizumab for the prevention of AMR in a group of 26 recipients with very high immunological risk [3]. These recipients who had a pretransplant positive crossmatch and DSA were treated with eculizumab in addition to plasmapheresis and thymoglobulin. When compared to a historical group (51 patients treated only with plasmapheresis and thymoglobulin), the incidence of AMR was significantly lower in patients treated with eculizumab (8% versus 41%).

Interestingly, at 1 year after transplant, the incidence of transplant glomerulopathy was also lower in the eculizumab group (7% versus 28%). Recently, Orandi et al. published a study using eculizumab alone or combined with splenectomy as rescue therapy for AMR. Of the 14 patients, 5 recipients treated with this combination had no graft loss. However, inquisitively, 80% of patients treated with eculizumab without splenectomy experienced graft loss [4].

In the literature, there are more than a dozen case reports on the use of eculizumab for treatment of AMR. In the majority of these cases, eculizumab is described as being effective in reversing the rejection and improving graft function [5–11]. However, in a minority of cases, eculizumab has failed to treat the rejection [12]. Interestingly, cases of cd4-negative AMR (including our case) do not seem to improve with eculizumab treatment. As c4d is a sign of complement activation, this suggests that there might be other mechanisms involved in AMR and eculizumab would be ineffective in this context. All cases are summarized in Table 1.

In kidney transplantation, eculizumab is an expensive treatment and its role in the treatment of antibody-mediated rejection remains to be determined.

## Figures and Tables

**Table 1:** Cases of antibody-mediated rejection treated with eculizumab.

	Patients	Regimen	Outcome
Locke et al. (2009) [5]	20-yo male Bone marrow transplant Positive DSA Positive crossmatch	1 dose (600 mg)	Improvement in allograft function and AMR resolution in biopsy

Lonze et al. (2010) [6]	43-yo female Kidney pair exchange Positive DSA Positive crossmatch	8 doses (1200 mg × 4, 600 mg × 4)	Normalization of allograft function

González-Roncero et al. (2012) [7]	2 cases 49-yo male and 34 yo female Low immunological risk	1 dose (600 mg)	Improvement in allograft function

Noone et al. (2012) [8]	13-yo female High immunological risk Factor H deficiency	2 doses (600 mg, 900 mg)	Improvement in allograft function

Stewart et al. (2012) [9]	29-yo male ABO incompatibility	5 doses	Improvement in allograft function and resolution of AMR in biopsy

Kocak et al. (2013) [10]	2 cases 26-yo female and 46-yo female Positive DSA	5 doses (5 × 900 mg)	Improvement in allograft function (only 1 recipient)

Burbach et al. (2014) [12]	2 cases 43-yo female and 36-yo male Positive DSA Negative C4d in biopsy	6 doses (900 mg × 4, 1200 mg × 2)	No improvement in allograft function

Chehade et al. (2015) [11]	7-yo male Positive DSA	2 doses (600 mg × 2)	Normalization of allograft function

Current case (2015)	50-yo female Pretransplant positive DSA Negative C4d in biopsy	3 doses (1200 mg × 1, 900 mg × 2)	Transient stabilization of allograft function Persistence of AMR in repeated biopsy

DSA: donor specific antibody.

yo: year-old.

AMR: antibody-mediated rejection.
